# Supplementary material for: Improving Nefiracetam Dissolution and Solubility Behavior Using a Cocrystallization Approach
Source: Pharmaceutics. 2020 Jul 9;12(7):653. doi: 10.3390/pharmaceutics12070653 (PMC7408141; doi:10.3390/pharmaceutics12070653)
Supplement: Supplementary file 1 [file pharmaceutics-12-00653-s001.pdf]

pharmaceutics

MDPI

# Supplementary Materials: Improving Nefiracetam Dissolution and Solubility Behavior Using a Cocrystallization Approach

**Xavier Buol, Koen Robeyns, Camila Caro Garrido, Nikolay Tumanov, Laurent Collard, Johan Wouters and Tom Leyssens**

## Cocrystal Screening

### *Coformer List*

**Table S1.** List of the coformers screened during the cocrystal screening. Suspected cocrystals (orange), Confirmed cocrystals (green) and no cocrystal (black).

| COFORMERS SCREENED | | |
|---|---|---|
| (L)-3-phenyllactic acid | (DL)-3-phenyllactic acid | (L)-ascorbic acid |
| (S)-2-phenylbutyric acid | (RS)-2-phenylbutyric acid | (RS)-2-phenoxypropionic acid |
| (RS)-2-phenoxypropionic acid | (RS)-2-phenoxypropionic acid | (RS)-oxiracetam |
| (S)-oxiracetam | (RS)-phenylsuccinic acid | (RS)-tropic acid |
| 1H-pyrazole-3,5-dicarboxylic acid monohydrate | 1-hydroxy-2-napthoic acid | 2,2-dimethylsuccinic acid |
| 2,3-dihydroxybenzoic acid | 2,4-dihydroxy benzoic acid | 2,5-dihydroxybenzoic acid |
| 2-aminobenzoic acid | 2-benzoylbenzoic acid | 2-hydroxy-1-napthoic acid |
| 2-ketoglutaric acid | 2-pyrrolidone-5-carboxylic acid | 3,4-dihydroxybenzoic acid |
| 3,5-dihydroxybenzoic acid | 3-aminobenzamide | 3-hydroxy-2-napthoic acid |
| 3-hydroxybenzoic acid | 3-methylbutanamide | 3-nitrobenzoic acid |
| (RS)-3-phenylbutyric acid | 4,4-bipyridine | 4-aminobenzamide |
| 4-aminobenzoic acid | 4-aminomethylbenzoic acid | 4-dimethylbenzoic acid |
| 4-hydroxybenzoic acid | 4-nitrobenzoic acid | 5-aminoisophthalic acid |
| 5-bromoisophthalic acid | 5-cyano-1,3-benzenedicarboxylic acid | 5-hydroxisophtalic acid |
| 5-methoxyisophthalic acid | 5-methylisophthalic acid | 5-nitroisophtalic acid |
| 6-hydroxy-2-napthoic acid | 7-(2-hydroxyethyl)theophylline | 7-(2-hydroxypropyll)theophylline |
| acetaminophen | acetoacetamide | acetylsalicylic acid |
| adipic acid | alanine (DL) | alanine (L) |
| aniracetam | anthranilamide | arginine (L) |
| asparagine (L) | aspartame | aspartic acid (DL) |
| aspartic acid (L) | benzenesulfonic acid | benzoic acid |
| calcium chloride | Caffeine | camphoric acid (D) |
| carphedon | carbamazepine | cholic acid |
| citraconic acid | citric acid | cysteine (L) |
| diphylline | (D)-mannitol | (D)-sorbitol |
| erythorbic acid | ethyl gallate | etiracetam |
| ferulic acid | flurbiprofen (RS) | fructose |
| fumaric acid | gallic acid | glucose |
| glutamine (L) | glutaric acid | glycine |
| lycolic acid | histidine (DL) | ibuprofen (RS) |
| isoleucine (DL) | isonicotinamide | isophtalic acid |
| ketoprofen (RS) | leucine (L) | leviteracetam |
| lysine (L) | maleic acid | malic acid |
| malonic acid | Maltol | mesaconic acid |

| | | |
|---|---|---|
| methionine (L) | methyl-3,4,5-trihydroxybenzoate | magnesium chloride |
| myo-inositol | naproxen (RS) | nicotinamide |
| nicotinic acid | oxalic acid | pamoic acid |
| p-coumaric acid | phenylalanine (DL) | phenylsulfoxide |
| phtalic acid | piracetam | pramiracetam |
| proline (DL) | proline (L) | pyridine-2,6-dicarboxylic acid |
| saccharin | salicylic acid | salicylic acid |
| serine (DL) | serine (L) | sorbic acid |
| stearic acid | succinic acid | sucrose |
| tartaric acid | theophylline | thiomalic acid |
| threonine (L) | trimellitic acid | trimesic acid |
| tryptophane (DL) | tryptophane (L) | tyrosine (L) |
| urea | valine (L) | xanthine |
| zinc chloride | | |

*PXRD patterns*

Suspected Cocrystals

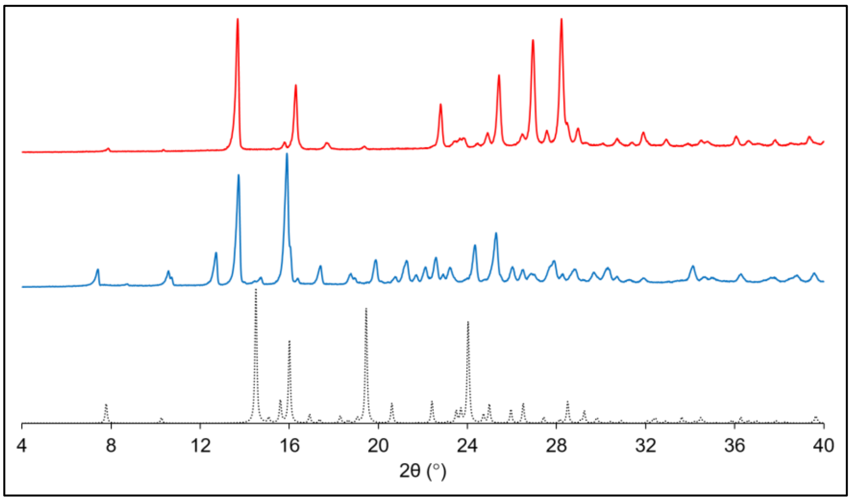


**Figure S1.** Experimental diffraction patterns of the 1:1 Nefiracetam-**2,4-dihydroxybenzoic acid** ground product (blue), the 2,4-dihydroxybenzoic acid coformer (red) and Nefiracetam simulated diffraction pattern (dashed black).

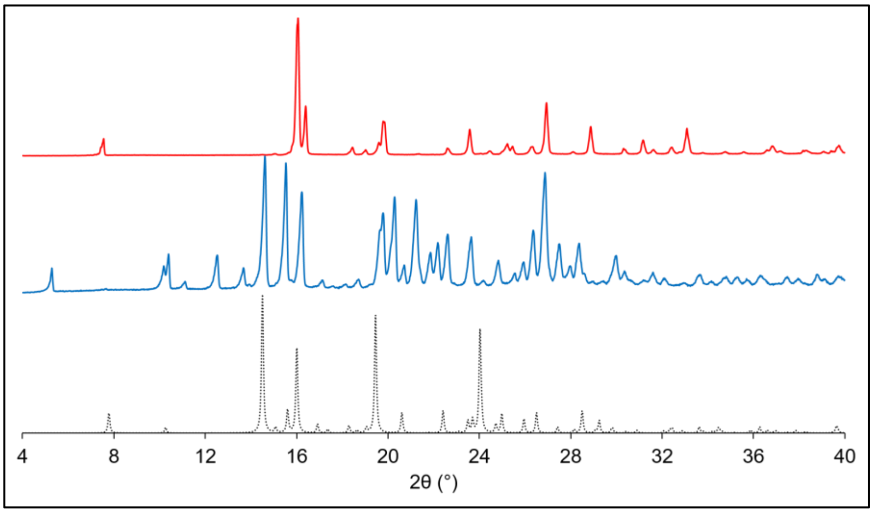


**Figure S2.** Experimental diffraction patterns of the 1:1 Nefiracetam-**2,5-dihydroxybenzoic acid** ground product (blue), the 2,5-dihydroxybenzoic acid coformer (red) and Nefiracetam simulated diffraction pattern (dashed black).

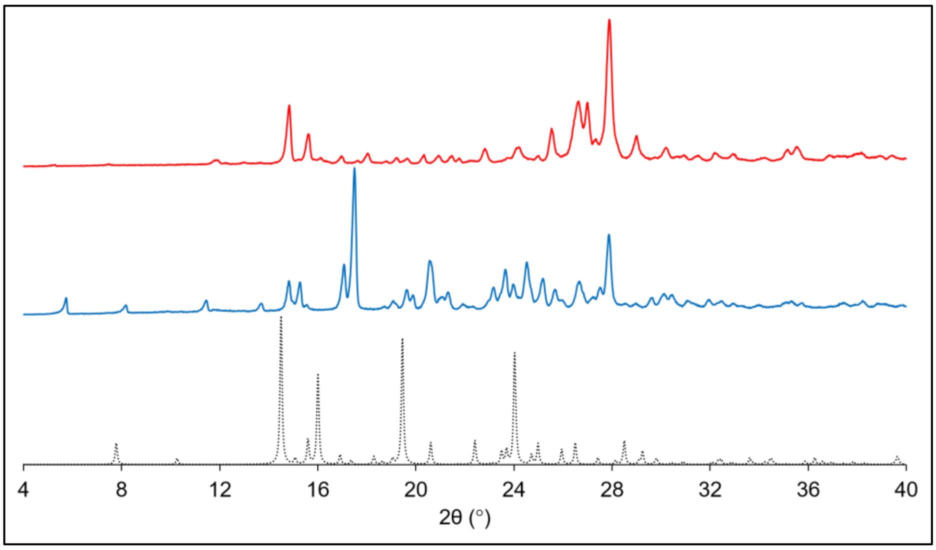


**Figure S3.** Experimental diffraction patterns of the 1:1 Nefiracetam-**3,4-dihydroxybenzoic acid** ground product (blue), the 3,4-dihydroxybenzoic acid coformer (red) and Nefiracetam simulated diffraction pattern (dashed black).

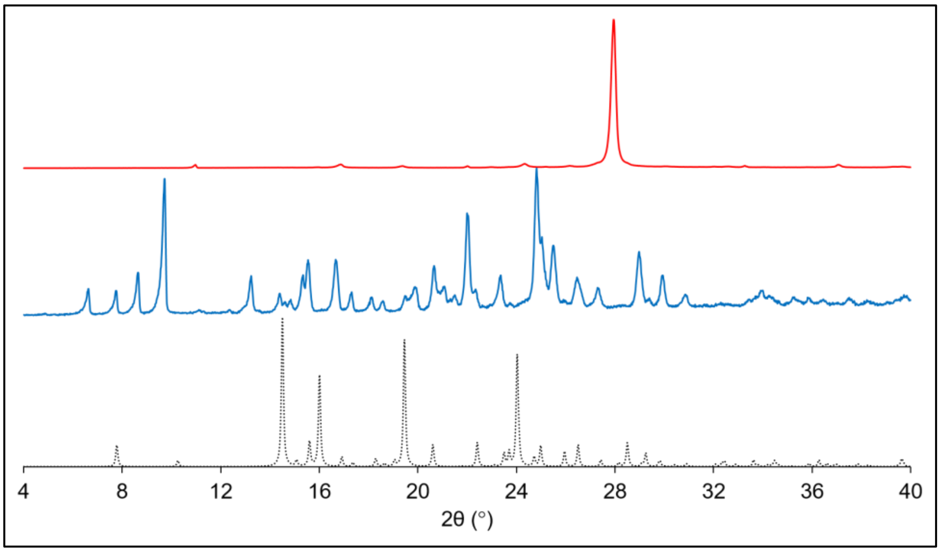


**Figure S4.** Experimental diffraction patterns of the 1:1 Nefiracetam-**pyridine-2,6-dicarboxylic acid** ground product (blue), the pyridine-2,6-dicarboxilic acid coformer (red) and Nefiracetam simulated diffraction pattern (dashed black).

Confirmed Cocrystals

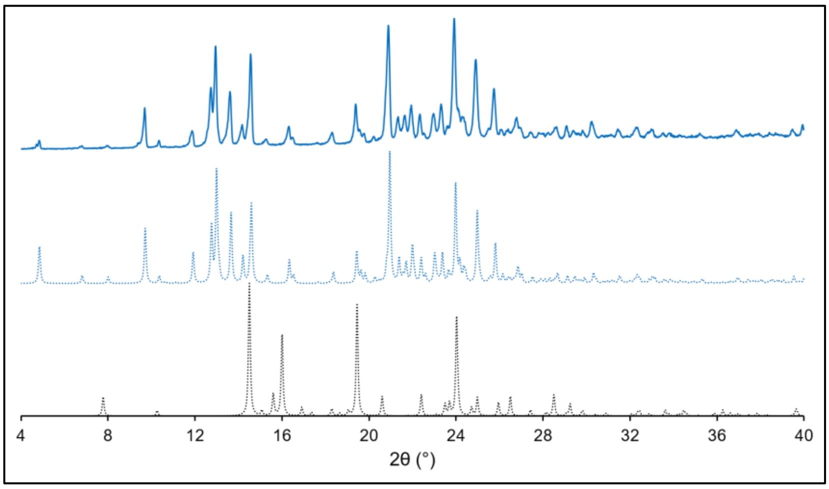


**Figure S5.** Simulated diffraction patterns (dashed line) of 1:1 **Nefiracetam-5-hydroxyisophthalic acid** cocrystal (blue) and comparison with the experimental one (full line) and the simulated diffractogram of Nefiracetam FI (black).

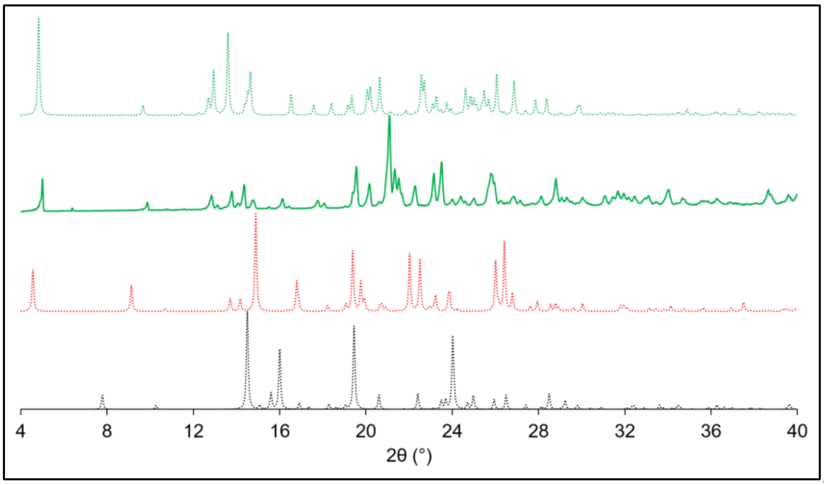


**Figure S6.** Simulated diffraction patterns (dashed line) of 1:1 **Nefiracetam-5-nitroisophthalic acid FI** cocrystal (green) and **FII** (red) and comparison with the experimental one (full line) and the simulated diffractogram of Nefiracetam FI (black).

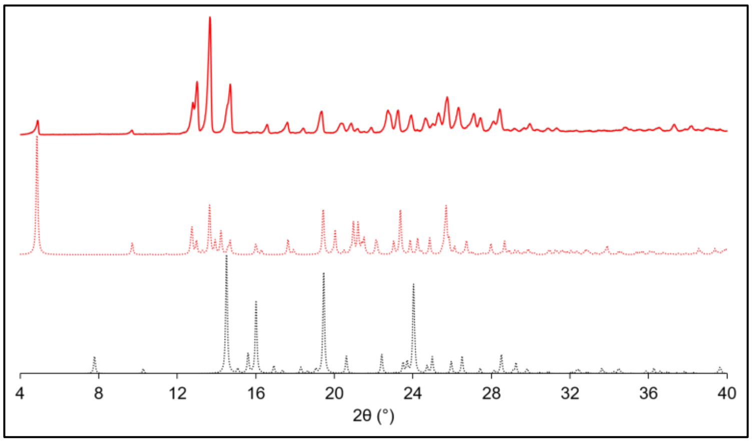


**Figure S7.** Simulated diffraction patterns (dashed line) of **Nefiracetam-5-bromoisophthalic acid** (red) and comparison with the experimental one (red full line) and the simulated diffractogram of Nefiracetam **FI** (black).

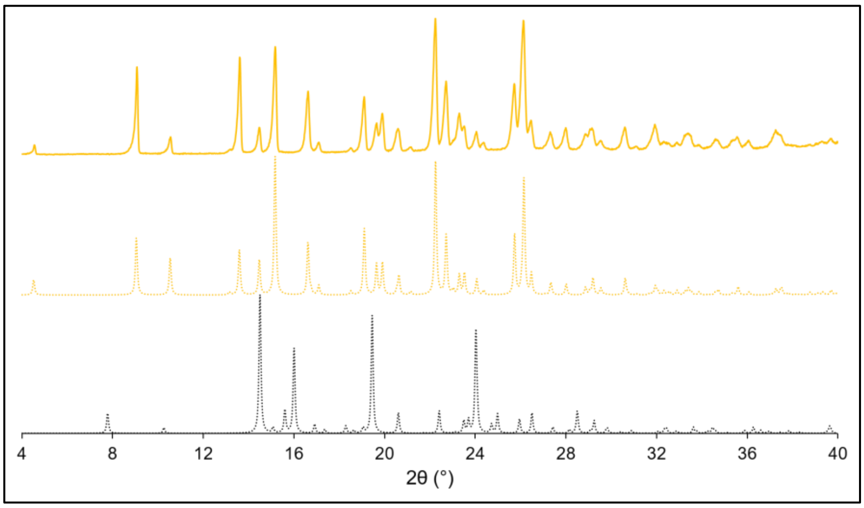


**Figure S8.** Simulated diffraction patterns (dashed line) of **Nefiracetam-5-cyano-1,3-benzenedicarboxylic acid** (orange) and comparison with the experimental one (full line) and the simulated diffractogram of Nefiracetam **FI** (black).

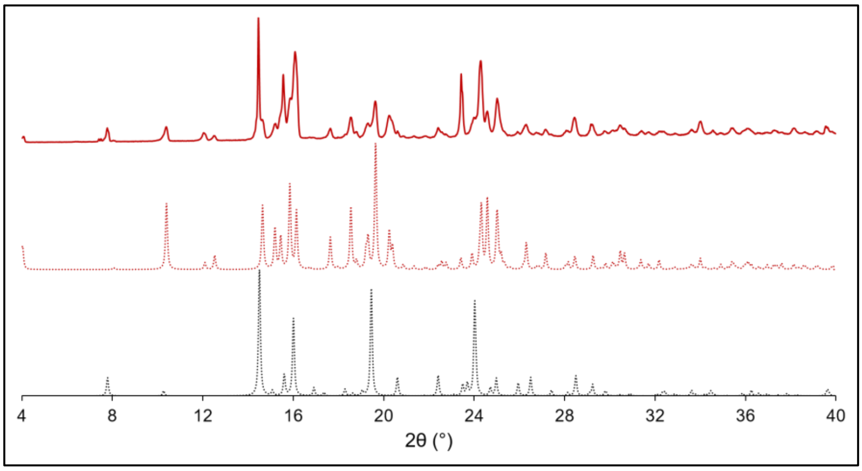


**Figure S9.** Simulated diffraction patterns (dashed line) of **Nefiracetam-2-benzylbenzoic acid** (dark red) and comparison with the experimental one (full line) and the simulated diffractogram of Nefiracetam FI (black).

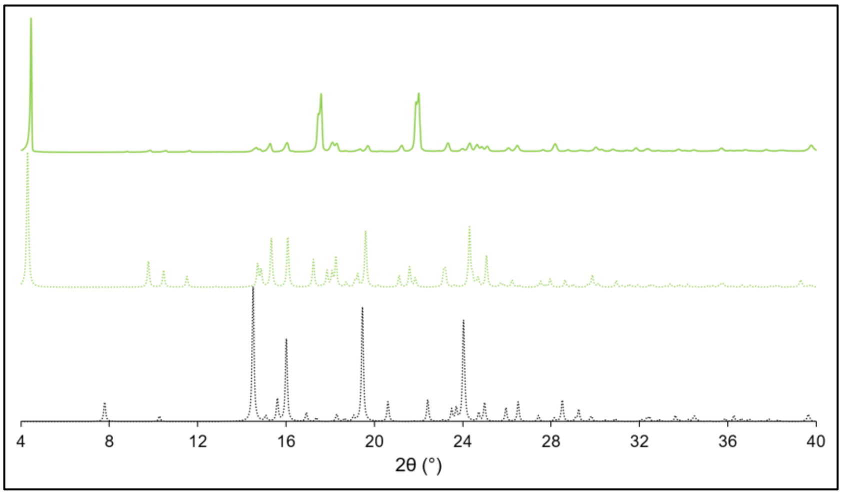


**Figure S10.** Simulated diffraction patterns (dashed line) of **Nefiracetam-2-phenylbutyric acid** cocrystal solid solution (light green) and comparison with the experimental one (full line) and the simulated diffractogram of Nefiracetam FI (black).

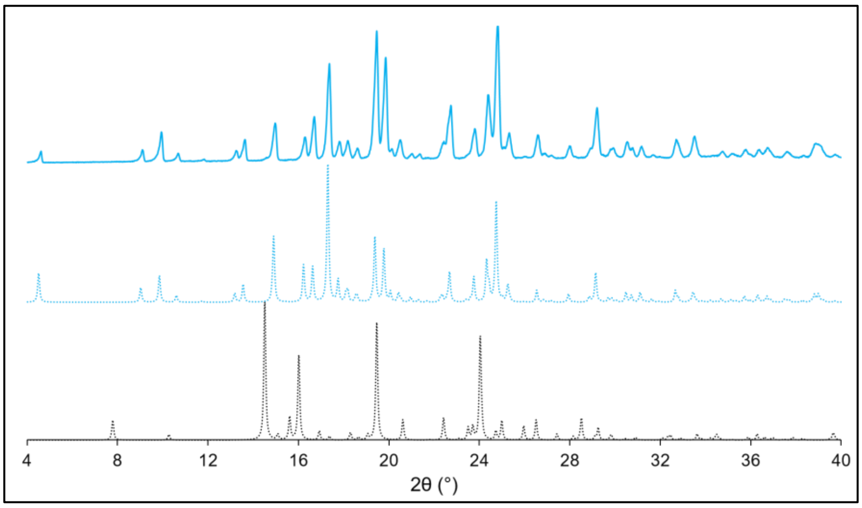


**Figure S11.** Simulated diffraction patterns (dashed line) of **Nefiracetam-(DL)-3-phenyllactic acid** racemic cocrystal (light blue) and comparison with the experimental one (full line) and the simulated diffractogram of Nefiracetam FI (black).

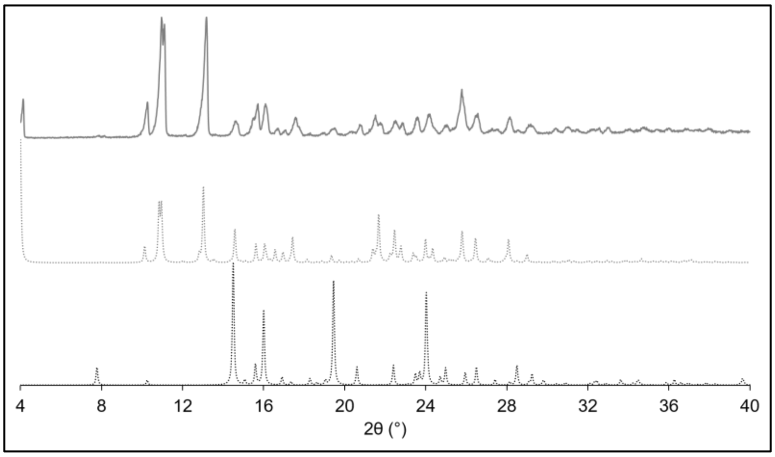


**Figure S12.** Simulated diffraction patterns (dashed line) of **4:1:1 Nefiracetam-3,4,5-trihydroxybenzoic acid (gallic acid)** cocrystal hydrate (grey) and comparison with the experimental one (full line) and the simulated diffractogram of Nefiracetam FI (black).

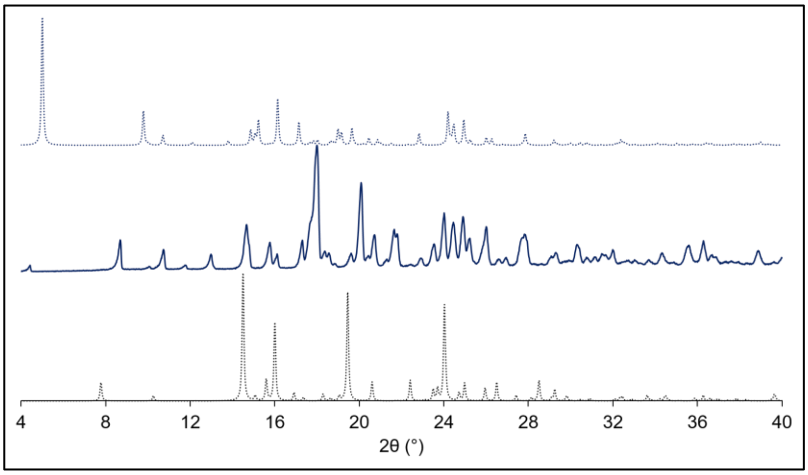


**Figure S13.** Simulated diffraction patterns (dashed line) of **2:1 Nefiracetam-(RS)-phenylsuccinic acid** racemic cocrystal (dark blue) and comparison with the ground product (full line) and the simulated diffractogram of Nefiracetam FI (black).

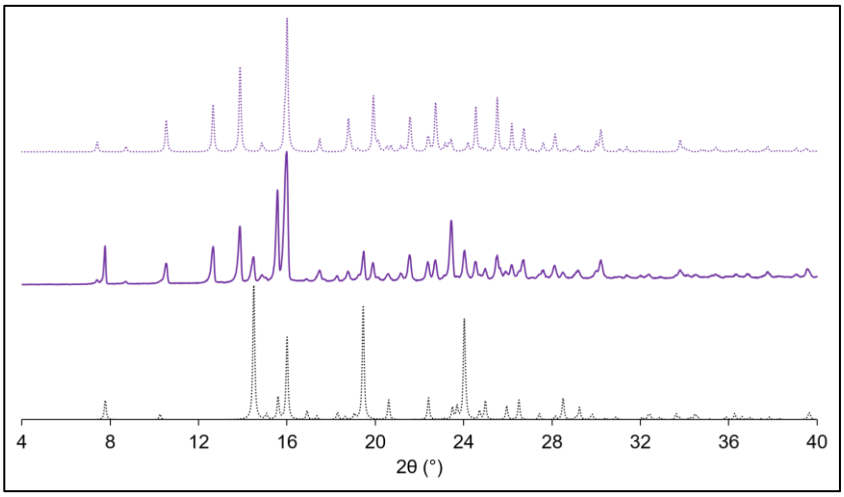


**Figure S14.** Simulated diffraction patterns (dashed line) of **1:1 Nefiracetam-4-hydrobenzoic acid** cocrystal (purple) and comparison with the ground product (full line) and the simulated diffractogram of Nefiracetam FI (black).

*DSC curves*

Suspected Cocrystals

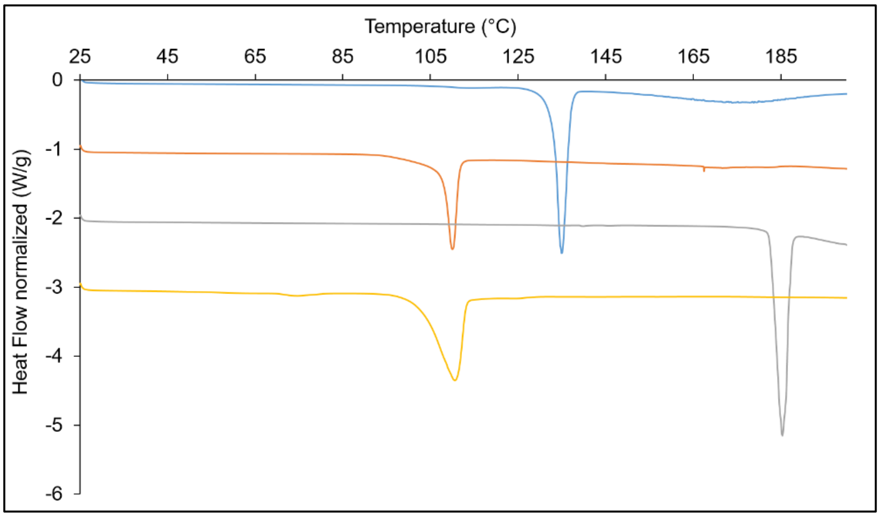


**Figure S15.** DSC curves of 1:1 Nefiracetam-**2,4-dihydroxybenzoic acid** (bleu), 1:1 Nefiracetam-**2,5-dihydroxybenzoic acid** (orange), 1:1 Nefiracetam-**pyridine-2,6-dicarboxylic acid** (grey) and 1:1 Nefiracetam-**3,4-dihydroxybenzoic acid** (gold) ground products.

Confirmed Cocrystals

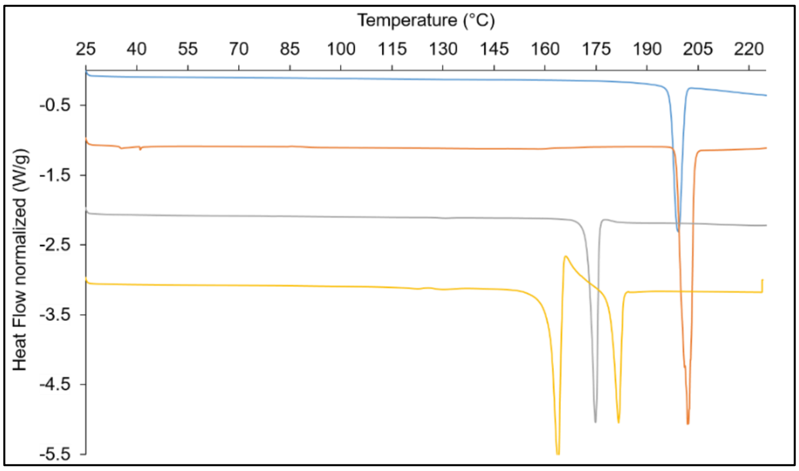


**Figure S16.** DSC curves of 1:1 Nefiracetam-5-hydroxyisophthalic acid (blue), 1:1 Nefiracetam-5-bromoisophthalic acid (orange), 1:1 Nefiracetam-5-nitroisophthalic acid FI (grey) and 1:1 Nefiracetam-5-cyano-1,3-benzendicarboxylic acid (gold) cocrystals.

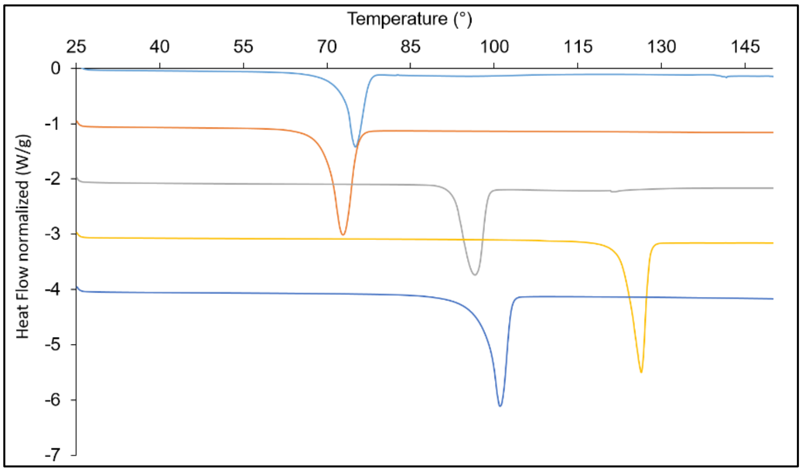


**Figure S17.** DSC curves of 1:1 Nefiracetam-(RS)-2-phenylbutyric acid (blue), 1:1 Nefiracetam-(DL)-3-phenyllactic acid (orange), and 1:1 Nefiracetam-2-benzoylbenzoic acid (grey), 1:1-Nefiracetam-4-hydroxybenzoic acid (gold) and 2:1 Nefiracetam-(RS)-phenylsuccinic acid (darker blue) cocrystals.

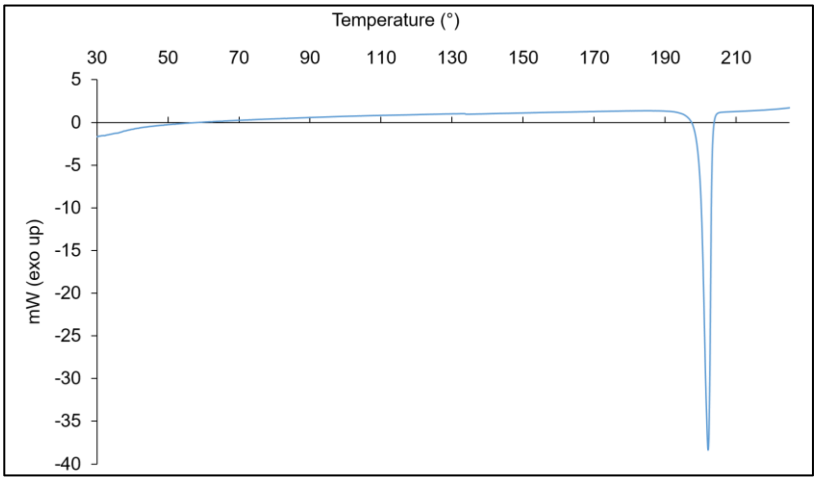


**Figure S18.** DSC curve of **the 1:1 Nefiracetam-5-nitroisophthalic acid FII cocrystal**. The measurement was occasionally performed on measurements were performed from 30°C to 225°C at a scanning rate of 5°C.min$^{-1}$ on a "Mettler Toledo DSC821e".

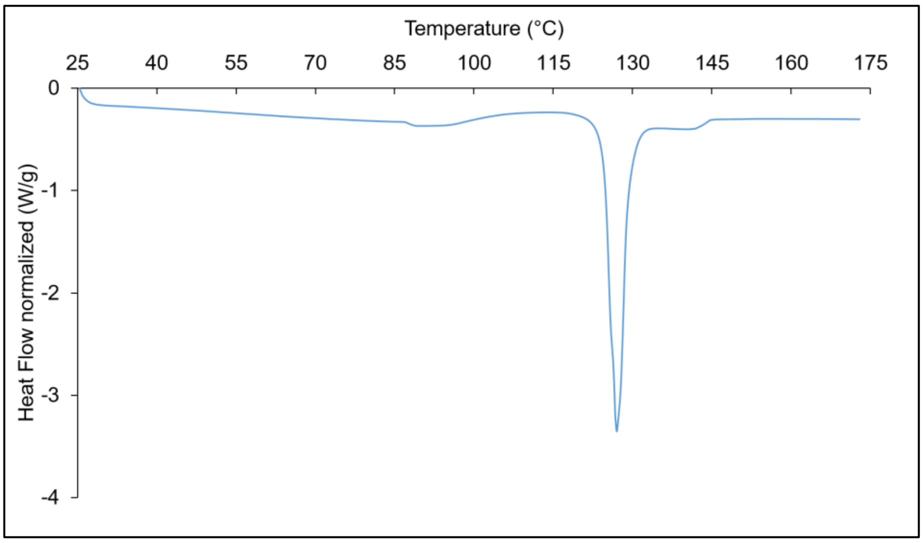


**Figure S19.** DSC curve of **4:1:1 Nefiracetam-gallic acid-water cocrystal hydrate**. Upon heating, the cocrystal decomposes into a mixture Nefiracetam and amorphous phase.

## Cocrystal Form Screening (NCA, NOA and NZC)

2:1 Nefiracetam-Citric Acid Cocrystal (NCA)

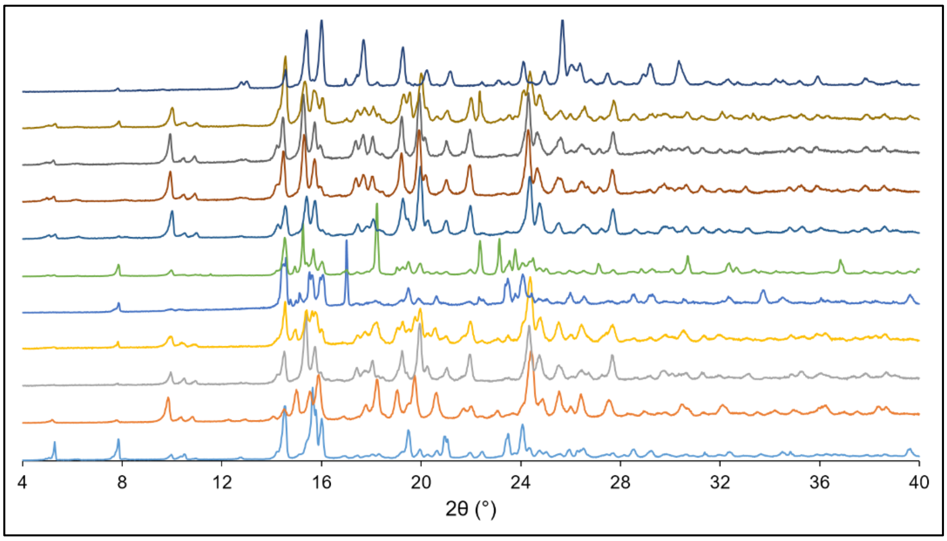


**Figure S20.** XRPD pattern on powders retrieved from the **NCA** form screening through **LAG** with different solvents. **1**: acetone; **2**: acetonitrile; **3**: chloroform; **4**: dichloromethane; **5**: ethyl acetate; **6**: ethanol; **7**: 2-propanol; **8**: methyl acetate; **9**: methanol; **10**: tetrahydrofuran; **11**: water.

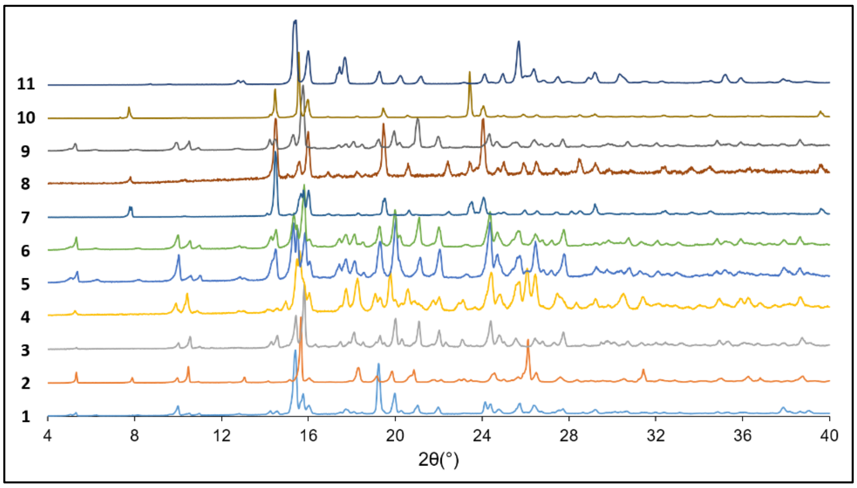


**Figure S21.** XRPD pattern on powders retrieved from the **NCA** form screening through **slurry crystallization** in different solvents. **1**: acetone; **2**: acetonitrile; **3**: chloroform; **4**: dichloromethane; **5**: diethyl ether; **6**: ethyl acetate; **7**: ethanol; **8**: 2-propanol; **9**: methyl acetate; **10**: tetrahydrofuran; **11**: water.

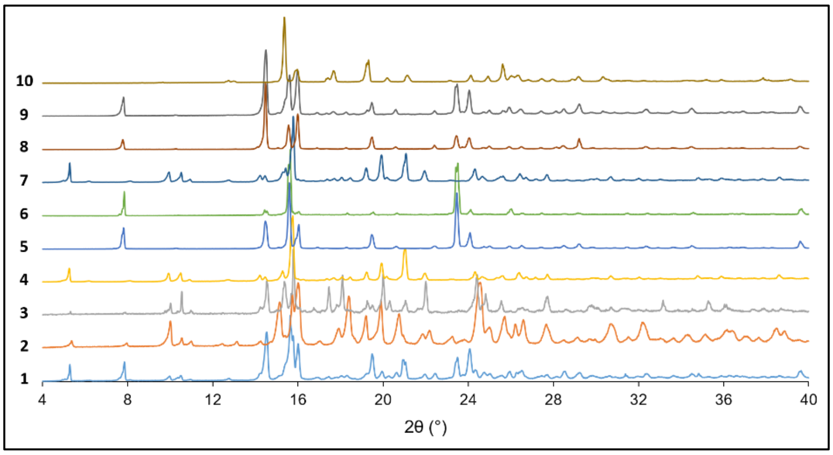


**Figure S22.** XRPD pattern on powders retrieved from the **NCA** form screening through **cooling crystallization** indifferent solvents. **1**: acetone; **2**: acetonitrile; **3**: chloroform; **4**: ethyl acetate; **5**: ethanol; **6**: 2-propanol; **7**: methyl acetate; **8**: methanol; **9**: tetrahydrofuran; **10**: water.

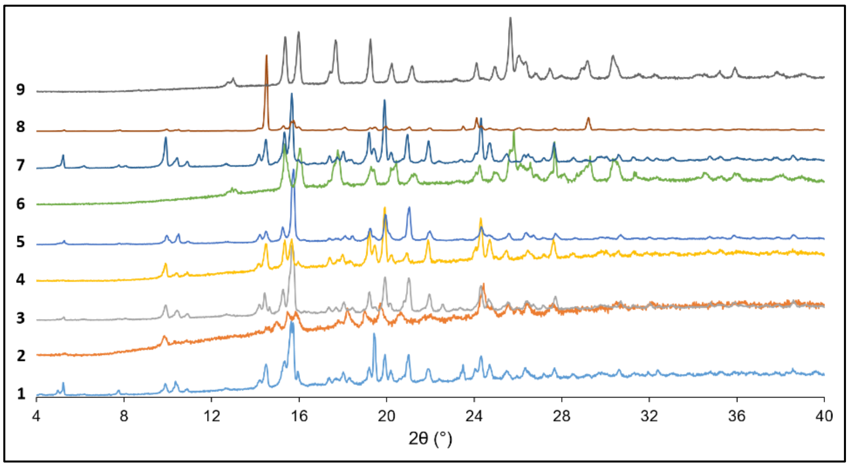


**Figure S23.** XRPD pattern on powders retrieved from the **NCA** form screening through **slow evaporation** in different solvents at room temperature. **1**: acetone; **2**: acetonitrile; **3**: ethyl acetate; **3**: ethanol; **5**: methyl acetate; **6**: methanol; **7**: 2-propanol; **8**: tetrahydrofuran; **9**: water.

2:1 Nefiracetam-Oxalic Acid Cocrystal (NOA)

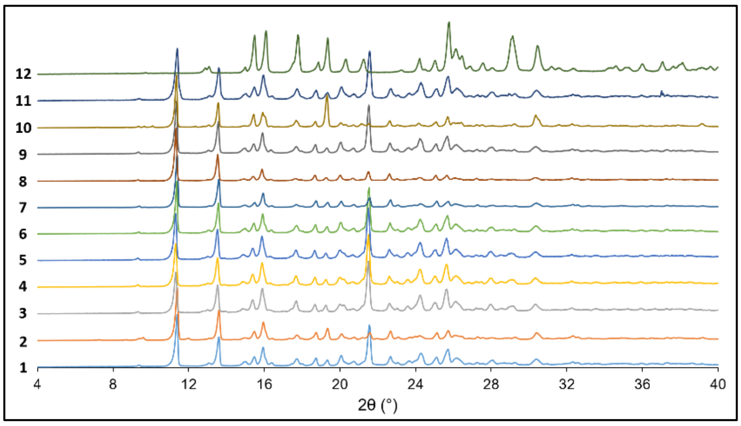


**Figure S24.** XRPD pattern on powders retrieved from the **NOA** form screening through **LAG** with different solvents. **1**: acetone; **2**: acetonitrile; **3**: chloroform; **4**: dichloromethane; **5**: diethyl ether; **6**: ethyl acetate; **7**: ethanol; **8**: 2-propanol; **9**: methyl acetate; **10**: methanol; **11**: tetrahydrofuran; **12**: water.

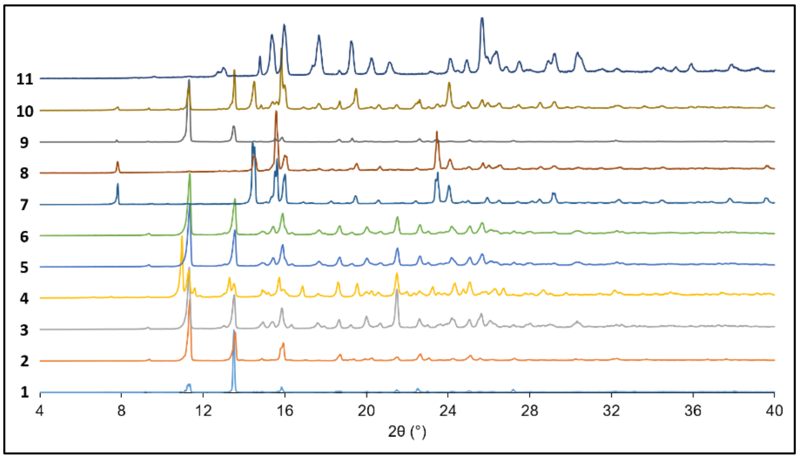


**Figure S25.** XRPD pattern on powders retrieved from the **NOA** form screening through **slurry crystallization** in different solvents at 25°C. **1**: acetone; **2**: acetonitrile; **3**: chloroform; **4**: dichloromethane; **5**: diethyl ether; **6**: ethyl acetate; **7**: ethanol; **8**: 2-propanol; **9**: methyl acetate; **10**: tetrahydrofuran; **11**: water.

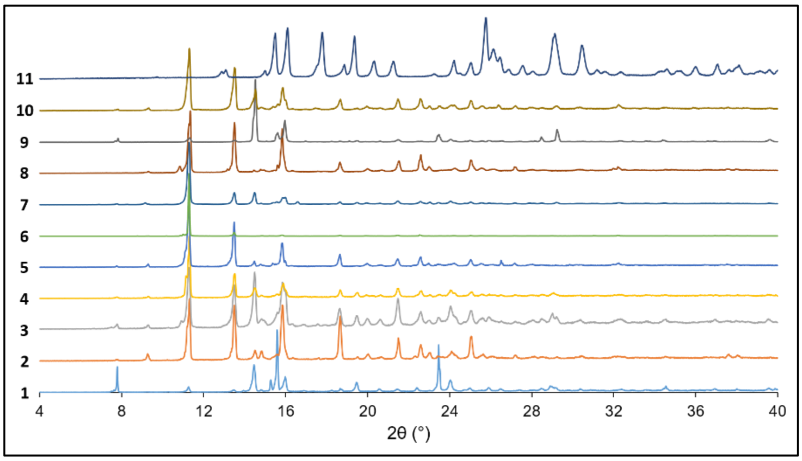


**Figure S26.** XRPD pattern on powders retrieved from the **NOA** form screening through **evaporative crystallization** in different solvents at room temperature. **1**: acetone; **2**: acetonitrile; **3**: chloroform; **4**: dichloromethane; **5**: ethyl acetate; **6**: ethanol; **7:** 2-propanol; **8**: methyl acetate; **9**: methanol; **10**: tetrahydrofuran; **11**: water.

1:1 Nefiracetam-Zinc Chloride Ionic Cocrystal (NZC)

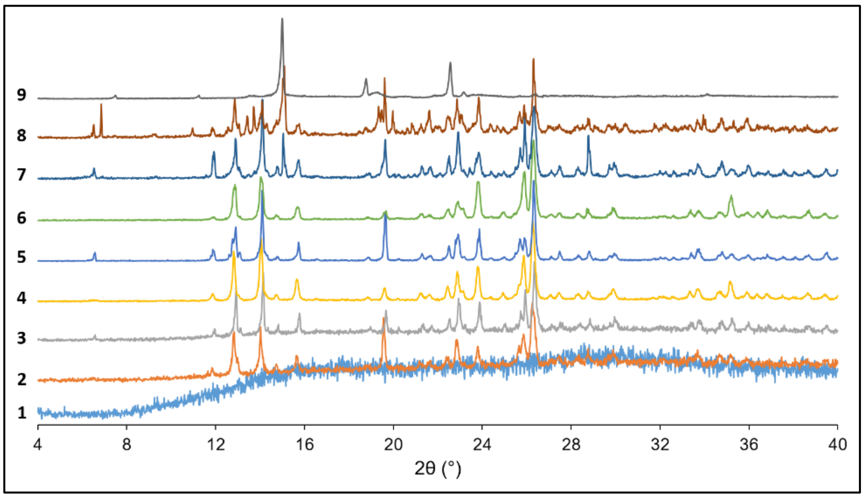


**Figure S27.** XRPD pattern on powders retrieved from the **NZC** stoichiometry screening through **slurry crystallization** in acetonitrile at 25°C. **1**: 100% ZC; **2**: ZC 89%-11% N; **3**: ZC 81%-19% N; **4**: ZC 70%-30% N; **5**: ZC 57%-43% N; **6**: ZC 48%-52% N; **7**: ZC 46%-54% N; **8**: ZC 30%-70% N; **9**: ZC 19%-81% N. Percent are given in molar ratio with N = Nefiracetam and ZC = zinc chloride.

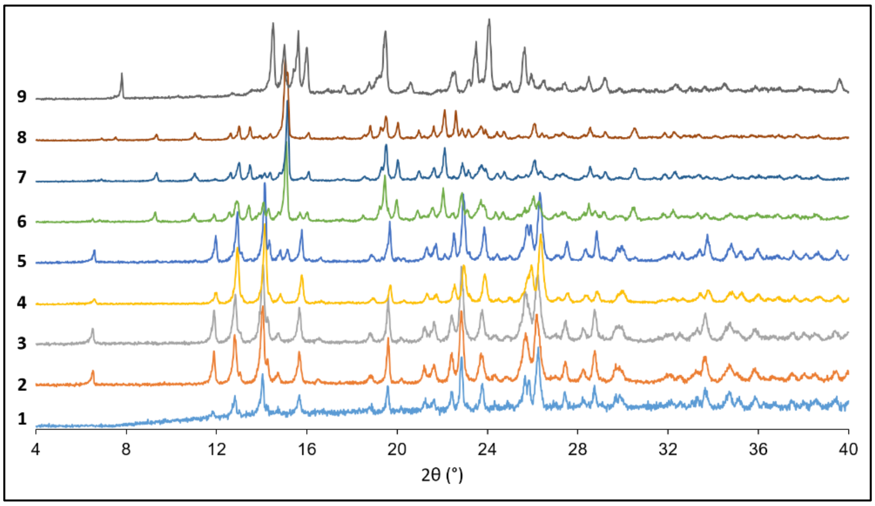


**Figure S28.** XRPD pattern on powders retrieved from the **NZC** stoichiometry screening through slurry crystallization in ethyl acetate at 25°C. **1**: ZC 89%-11% N; **2**: ZC 80%-20% N; **3**: ZC 68%-32% N; **4**: ZC 59%-41% N; **5**: ZC 50%-50% N; **6**: ZC 39%-61% N; **7**: ZC 37%-63% N; **8**: ZC 31%-69% N; **9**: ZC 10%-90% N. Percent are given in molar ratio with N = Nefiracetam and ZC = zinc chloride.

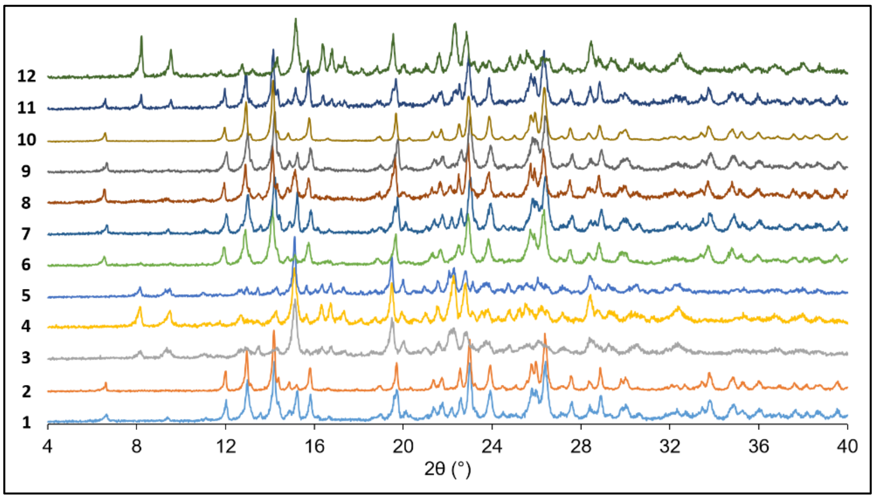


**Figure S29.** XRPD pattern on powders retrieved from the **NZC** form screening through LAG with different solvents. **1**: acetone; **2**: acetonitrile; **3**: chloroform; **4**: dichloromethane; **5**: diethyl ether; **6**: ethyl acetate; **7**: ethanol; **8**: methyl acetate; **9**: 2-propanol; **10**: methanol; **11**: tetrahydrofuran ; **12**: water.

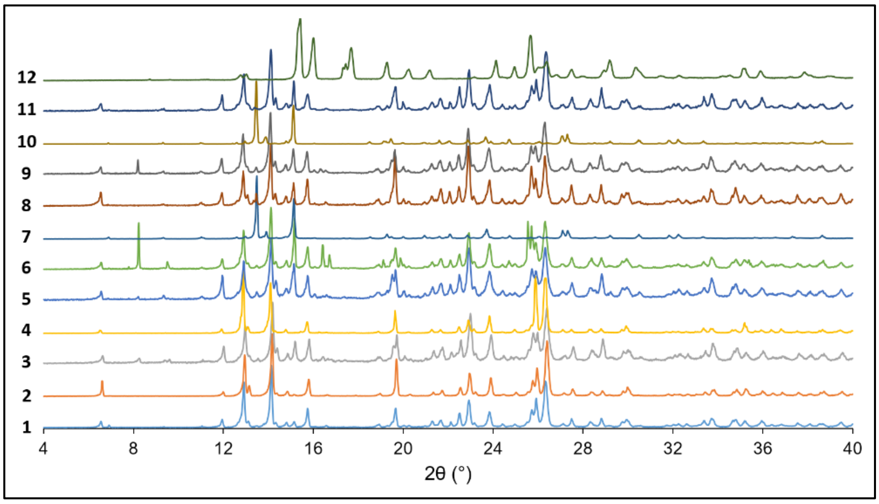


**Figure S30.** XRPD pattern on powders retrieved from the **NZC** form screening through **slurry crystallization** with different solvents at 25°C. **1**: acetone; **2**: acetonitrile; **3**: chloroform; **4**: dichloromethane; **5**: diethyl ether; **6**: ethyl acetate; **7**: ethanol; **8**: 2-propanol; **9**: methyl acetate; **10**: methanol; **11**: tetrahydrofuran; **12**: water.

## Crystal Packing and Crystallographic Tables (other structures)

CCDC 2010261-2010276 contain the supplementary crystallographic data for this paper. These data can be obtained free of charge from The Cambridge Crystallographic Data Centre via www.ccdc.cam.ac.uk/structures

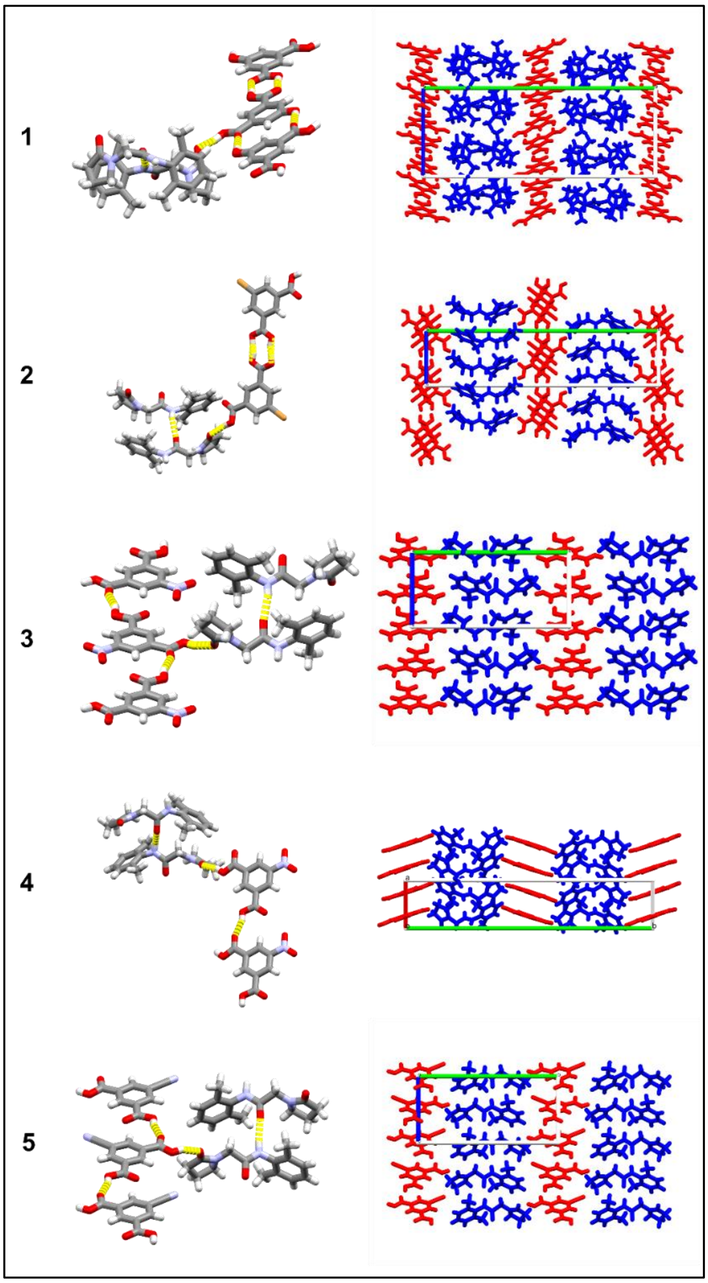


**Figure S31.** (left) Main intermolecular interactions and (right) crystal packing of **1**: 1:1 Nefiracetam-5-hydroxyisophthalic acid, **2**: 1:1 Nefiracetam-5-bromoisophthalic acid, **3**: 1:1 Nefiracetam-5-nitroisophthalic acid FII, **4**: 1:1 Nefiracetam-5-nitroisophthalic acid FI and **5**: 1:1 Nefiracetam-5-cyano-1,3-benzenedicarboxylic acid cocrystals. The Nefiracetam molecules and coformers are respectively colored in blue and red in the crystal lattices. Yellow contact sticks are used to highlight the intermolecular hydrogen bonds.

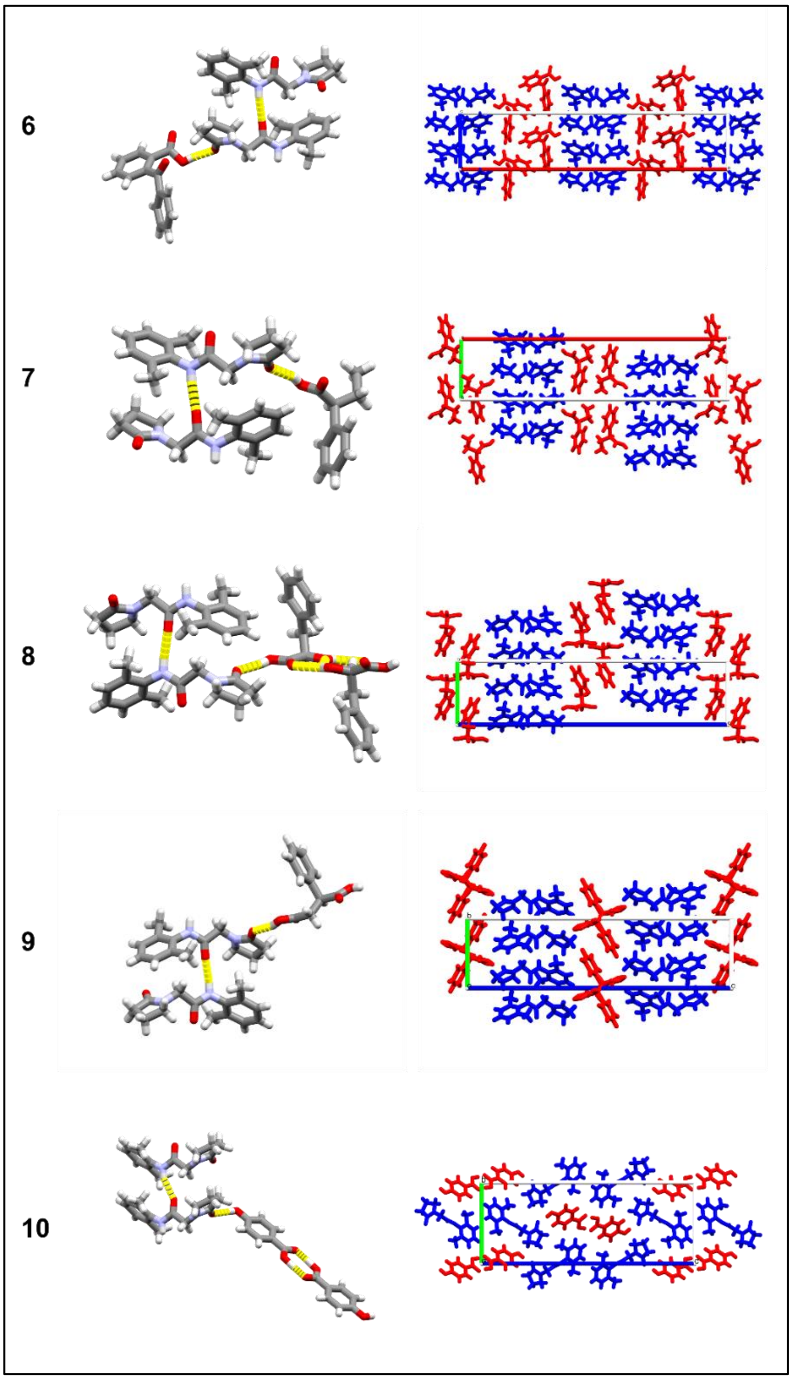


**Figure S32.** (left) Main intermolecular interactions and (right) crystal packing of **6**: 1:1 Nefiracetam-2-benzoyl benzoic acid, **7**: Nefiracetam-(RS)-2-phenylbutyric acid and **8**: Nefiracetam-(DL)-3-phenyllactic acid (solid solution), **9**: 2:1 Nefiracetam-(RS)-phenylsuccinic acid (racemic) and **10**: 1:1 Nefiracetam-4-hydroxybenzoic acid cocrystals. The Nefiracetam molecules and coformers are respectively colored in blue and red in the crystal lattices. Yellow contact sticks are used to highlight the intermolecular hydrogen bonds. The cocrystal structure of 2:1 Nefiracetam-(RS)-phenylsuccinic acid exhibits an inversion centre on each phenylsuccinic acid molecule.

**Table S2.** Main crystallographic data of **NCA**, **NCA1**, **NOA**, **NZC** and **NZCW** and refinement parameters.

| Compound | NCA | NCA1 | NOA | NZC | NZCW |
|---|---|---|---|---|---|
| Empirical formula | $C_{68}H_{87}N_8O_{22}$ | $C_{34}H_{43}N_{24}O_{11}$ | $C_{30}H_{38}N_4O_8$ | $C_{14}H_{18}Cl_2N_2O_2Zn$ | $C_{14}H_{20}Cl_2N_2O_3Zn$ |
| Formula weight (g.mol$^{-1}$) | 1368.45 | 683.72 | 582.84 | 382.57 | 400.59 |
| Temperature (K) | 297(2) | 297(2) | 100(2) | 100(2) | 297(2) |
| Wavelength (Å) | 1.54184 | 1.54184 | 0.798 | 0.71073 | 0.71073 |
| Crystal system | Triclinic | Monoclinic | Monoclinic | Monoclinic | Monoclinic |
| Space group | *P*-1 | *P*21/*c* | *P*2$_1$ | *C*2/*c* | *P*2$_1$/*c* |
| a, b, c (Å) | 6.1877(2),18.7399(4),35.3540(11) | 6.439(3),35.361(9),9.782(3) | 88.4663(5),15.4852(9),23.4755(11) | 14.9275(3),7.66202(16),27.3619(6) | 8.8632(3),18.2415(8),22.1746(12) |
| α, β, γ (°) | 74.626(3), 87.939(3), 82.248(2) | 90, 97.68(3). 90 | 90, 99.903(6), 90 | 90, 97.3147(19), 90 | 90, 101.353(5), 90 |
| Volume (Å$^3$) | 3916.7(2) | 2207.3(13) | 3031.8(3) | 3104.06(11) | 3515.0(3) |
| Z | 2 | 2 | 4 | 8 | 8 |
| Density (g.cm$^{-3}$) | 1.160 | 1.029 | 1.276 | 1.637 | 1.514 |
| Absorption coefficient (mm$^{-1}$) | 0.727 | 0.645 | 0.120 | 1.931 | 1.713 |
| F(000) | 1454 | 726 | 1240 | 1568 | 1648 |
| Crystal size (mm$^3$) | 0.500 x 0.385 x 0.039 | Crystallized on **NCA** | 0.50 x 0.04 x 0.02 | 0.490 x 0.280 x 0.280 | 0.45 x 0.30 x 0.07 |
| Theta range for data collection (°) | 6.493 to 66.601 | 8.182 to 58.930 | 1.777 to 28.729 | 2.751 to 32.666 | 3.025 to 25.688 |
| Reflections collected | 13586 | 14428 | 24245 | 10307 | 31528 |
| Completeness to θ = 25.242° | 98.8 % (to theta = 66.601°) | 99% | 98.1 % (to 28.607°) | 100.0 % | 99.7 % |
| Max. and min. transmission | 1.000 and 0.836 | 1.000 and 0.836 | 1.000 and 0.747 | 0.701 and 0.582 | 1.000 and 0.428 |
| Refinement method | Full-matrix least-squares on F$^2$ | Full-matrix least-squares on F$^2$ | Full-matrix least-squares on F$^2$ | Full-matrix least-squares on F$^2$ | Full-matrix least-squares on F$^2$ |
| Data / restraints / parameters | 13586 / 451 / 972 | 3125 / 30 / 282 | 24245 / 1 / 767 | 5151 / 0 / 196 | 6638 / 0 / 407 |
| Goodness-of-fit on F$^2$ | 1.086 | 1.087 | 1.091 | 1.105 | 1.037 |
| Final R indices [I>2sigma(I)] | R1 = 0.0747, wR2 = 0.1703 | R1 = 0.0969, wR2 = 0.1878 | R1 = 0.0806, wR2 = 0.2053 | R1 = 0.0258, wR2 = 0.0577 | R1 = 0.0351, wR2 = 0.0846 |
| R indices (all data) | R1 = 0.0849, wR2 = 0.1777 | R1 = 0.1688, wR2 = 0.2275 | R1 = 0.0976, wR2 = 0.2213 | R1 = 0.0295, wR2 = 0.0590 | R1 = 0.0447, wR2 = 0.0891 |
| Δϱ (max, min) (e.Å$^{-3}$) | 0.498 and -0.274 | 0.238 and -0.201 | 0.312 and -0.2451 | 0.427 and -0.395 | 0.541 and -0.411 |

**Table S3.** Main crystallographic data and refinement parameters of all cocrystal structures (part 1) not presented in the main manuscript.

| Compound | 1:1 Nefiracetam-5-nitroisophthalic acid FI | 1:1 Nefiracetam-5-nitroisophthalic acid FII | 1:1 Nefiracetam-5-bromoisophthalic acid | 1:1 Nefiracetam-5-hydroxyisophthalic acid |
|---|---|---|---|---|
| Empirical formula | $C_{22} H_{23} N_3 O_8$ | $C_{22} H_{23} N_3 O_8$ | $C_{22} H_{23} Br N_2 O_6$ | $C_{22} H_{24} N_2 O_7$ |
| Formula weight (g.mol$^{-1}$) | 457.43 | 457.43 | 491.33 | 428.43 |
| Temperature (K) | 297(2) | 295(2) | 297(2) | 297(2) |
| Wavelength (Å) | 1.54184 | 1.54184 | 0.71073 | 0.71073 |
| Crystal system | Monoclinic | Monoclinic | Monoclinic | Monoclinic |
| Space group | $P2_1/n$ | $Pc$ | $P2_1/c$ | $P2_1/c$ |
| a, b, c (Å) | 7.87163(18),36.5803(6),8.70817(18) | 6.33727(5),19.36454(12), 9.28378(8) | 7.5009(5), 6.4477(18), 9.2134(5) | 8.7567(4), 36.4005(17), 14.2304(6) |
| α, β, γ (°) | 90, 115.254(3), 90 | 90, 99.8062(8), 90 | 90, 112.191 (8), 90 | 90, 102.985(4), 90 |
| Volume (Å$^3$) | 2267.84(9) | 1122.644(14) | 2332.3(3) | 4419.9(3) |
| Z | 4 | 2 | 4 | 8 |
| Density (g.cm$^{-3}$) | 1.340 | 1.353 | 1.399 | 1.288 |
| Absorption coefficient (mm$^{-1}$) | 0.872 | 0.881 | 1.801 | 0.0997 |
| F(000) | 960 | 480 | 1008 | 1808 |
| Crystal size (mm$^3$) | 0.572 x 0.423 x 0.239 | 0.450 x 0.330 x 0.047 | 0.45 x 0.30 x 0.10 | 0.50 x 0.50 x 0.40 |
| θ range for data collection (°) | 4.836 to 67.118 | 2.282 to 67.115 | 3.439 to 25.790 | 2.918 to 26.267 |
| Reflections collected | 11946 | 24140 | 10325 | 31147 |
| Completeness (%) | 98.6 % (to theta = 67.118°) | 99.5 (to theta = 67.115°) | 96.8 (to theta = 25.242°) | 99.0 (to theta = 25.242°) |
| Max. and min. transmission | 1.000 and 0.918 | 0.960 and 0.763 | 1.000 and 0.705 | 1.000 and 0.873 |
| Refinement method | Full-matrix least-squares on $F^2$ | Full-matrix least-squares on $F^2$ | Full-matrix least-squares on $F^2$ | Full-matrix least-squares on $F^2$ |
| Data / restraints / parameters | 4012 / 147 / 441 | 3871 / 2 / 306 | 4295 / 5 / 320 | 8765 / 0 / 571 |
| Goodness-of-fit on $F^2$ | 1.088 | 0.950 | 1.082 | 1.056 |
| Final R indices [I>2sigma(I)] | R1 = 0.0465, wR2 = 0.1153 | R1 = 0.0295, wR2 = 0.0885 | R1 = 0.0515, wR2 = 0.1367 | R1 = 0.0516, wR2 = 0.1404 |
| R indices (all data) | R1 = 0.0510, wR2 = 0.1191 | R1 = 0.0298, wR2 = 0.0892 | R1 = 0.0767, wR2 = 0.1536 | R1 = 0.0613, wR2 = 0.1477 |
| Δρ (max, min) (e.Å$^{-3}$) | 0.216 and -0.172 | 0.149 and -0.146 | 0.354 and -0.426 | 0.262 and -0.187 |

**Table S4.** Main crystallographic data and refinement parameters of all cocrystal structures (part 2) not presented in the main manuscript.

| Compound | 1:1 Nefiracetam-5-cyano-1,3-benzenedicarboxylic | Nefiracetam-(RS)-2-phenylbutyric acid | 1:1 Nefiracetam-2-benzoylbenzoic acid | 1:1 Nefiracetam-(RS)-3-phenyllactic acid |
|---|---|---|---|---|
| Empirical formula | $C_{23} H_{23} N_3 O_6$ | $C_{24} H_{30} N_2 O_4$ | $C_{28} H_{28} N_2 O_5$ | $C_{23} H_{28} N_2 O_5$ |
| Formula weight (g.mol$^{-1}$) | 437.44 | 410.50 | 472.52 | 412.47 |
| Temperature (K) | 297(2) | 297(2) K | 297(2) | 297(2) K |

| | | | | |
|---|---|---|---|---|
| Wavelength (Å) | 0.71073 | 0.71073 Å | 0.71073 | 0.71073 Å |
| Crystal system | Monoclinic | Orthorhombic | Orthorhombic | Orthorhombic |
| Space group | *Pc* | $P2_12_12$ | *Pca*21 | $P2_12_12_1$ |
| a, b, c (Å) | 6.1863(3), 19.5370(8), 9.3910(4) | 41.158(2), 9.2846(6), 6.0202(4) | 43.8966(14),6.04575(19),9.2293(3) | 6.0151(5), 9.2249(6), 39.209(2) |
| α, β, γ (°) | 90, 98.507, 90 | 90, 90, 90 | 90, 90, 90 | 90, 90, 90 |
| Volume (Å$^3$) | 1122.52(8) | 2300.5(3) | 2449.33(13) | 2175.6(3) Å$^3$ |
| Z | 2 | 4 | 4 | 4 |
| Density (g.cm$^{-3}$) | 1.294 | 1.185 Mg/m$^3$ | 1.281 | 1.259 Mg/m$^3$ |
| Absorption coefficient (mm$^{-1}$) | 0.095 | 0.081 mm$^{-1}$ | 0.088 | 0.089 mm$^{-1}$ |
| F(000) | 460 | 880 | 1000 | 880 |
| Crystal size (mm³) | 0.35 x 0.28 x 0.09 | 0.490 x 0.301 x 0.148 | 0.28 x 0.19 x 0.02 | 0.410 x 0.270 x 0.085 |
| Theta range for data collection (°) | 3.026 to 25.243 | 2.249 to 25.679° | 3.402 to 25.265 | 2.268 to 25.247°. |
| Reflections collected | 7577 | 6688 | 19309 | 5814 |
| Completeness (%) | 94.9 (to theta = 25.242°) | 99.5 % | 99.2 (to theta = 25.242°) | 99.0 % |
| Max. and min. transmission | 1.00000 and 0.85329 | 0.959 and 0.871 | 1.00000 and 0.88720 | 0.975 and 0.923 |
| Refinement method | Full-matrix least-squares on F$^2$ | Full-matrix least-squares on F$^2$ | Full-matrix least-squares on F$^2$ | Full-matrix least-squares on F$^2$ |
| Data / restraints / parameters | 7577 / 2 / 294 | 4063 / 81 / 360 | 4399 / 1 / 318 | 3606 / 0 / 275 |
| Goodness-of-fit on F$^2$ | 1.040 | 1.082 | 1.077 | 1.057 |
| Final R indices [I>2sigma(I)] | R1 = 0.0404, wR2 = 0.1028 | R1 = 0.0667, wR2 = 0.1123 | R1 = 0.0442, wR2 = 0.1051 | R1 = 0.0619, wR2 = 0.1027 |
| R indices (all data) | R1 = 0.0589, wR2 = 0.1084 | R1 = 0.1118, wR2 = 0.1320 | R1 = 0.0514, wR2 = 0.1093 | R1 = 0.0915, wR2 = 0.1211 |
| Δρ (max, min) (e.Å$^{-3}$) | 0.135 and -0.110 | 0.148 and -0.148 | 0.188 and -0.161 | 0.164 and -0.166 |

**Table S5.** Main crystallographic data and refinement parameters of all cocrystal structures (part 3) not presented in the main manuscript.

| Compound | 2:1 Nefiracetam-(RS)-phenylsuccinic acid | 1:1 Nefiracetam-4-hydroxybenzoic acid | 4:1:1 Nefiracetam-gallic acid-water |
|---|---|---|---|
| Empirical formula | $C_{76}H_{92}N_8O_{16}$ | $C_{21}H_{24}N_2O_5$ | $C_{64}H_{80}N_8O_{14}$ |
| Formula weight (g.mol$^{-1}$) | 1373.57 | 384.42 | 1185.36 |
| Temperature (K) | 293(2) | 295(2) | 293(2) |
| Wavelength (Å) | 1.54184 | 1.54184 | 0.71073 |
| Crystal system | Monoclinic | Monoclinic | Triclinic |
| Space group | $P2_1/n$ | $P2_1/n$ | *P*-1 |
| a, b, c (Å) | 5.98623(16), 9.3427(2), 35.3554(9) | 4.7571(2), 12.7544(5), 33.4306(15) | 8.3372(5), 8.8647(12), 22.302(2) |
| α, β, γ (°) | 90, 94.247(3), 90 | 90, 91.108(4), 90 | 87.663(10), 81.860 (7), 79.777(8) |
| Volume (Å$^3$) | 1971.91(9) | 2027.99(15) | 1605.5(3) |
| Z | 1 | 4 | 1 |
| Density (g.cm$^3$) | 1.157 | 1.259 | 1.226 |
| Absorption coefficient (mm$^{-1}$) | 0.666 | 0.744 | 0.087 |
| F(000) | 732 | 816 | 632 |
| Crystal size (mm³) | 0.533 x 0.333 x 0.270 | 0.480 x 0.350 x 0.240 | 0.55 x 0.10 x 0.06 |
| θ range for data collection (°) | 5.017 to 66.872° | 2.644 to 67.194 | 2.902 to 25.090° |

| | | | |
|---|---|---|---|
| Reflections collected | 11005 | 5363 | 16276 |
| Completeness (%) | 98.0 % (to theta = 66.872°) | 98.4 % (to theta = 67.194°) | 98.9% (to theta = 25.090°) |
| Max. and min. transmission | 1.000 and 0.918 | 1.000 and 0.938 | 1.000 and 0.765 |
| Refinement method | Full-matrix least-squares on $F^2$ | Full-matrix least-squares on $F^2$ | Full-matrix least-squares on $F^2$ |
| Data / restraints / parameters | 3431 / 62 / 273 | 5363 / 0 / 264 | 5650 / 75 / 437 |
| Goodness-of-fit on $F^2$ | 1.088 | 1.093 | 1.096 |
| Final R indices [I>2sigma(I)] | R1 = 0.0799, wR2 = 0.1873 | R1 = 0.0439, wR2 = 0.1385 | R1 = 0.0666, wR2 = 0.1601 |
| R indices (all data) | R1 = 0.0830, wR2 = 0.1889 | R1 = 0.0547, wR2 = 0.1423 | R1 = 0.0985, wR2 = 0.1765 |
| Largest diff. peak and hole (e.Å$^{-3}$) | 0.623 and -0.351 | 0.176 and -0.152 | 0.261 and -0.330 |

## DVS and Moisture Exposure

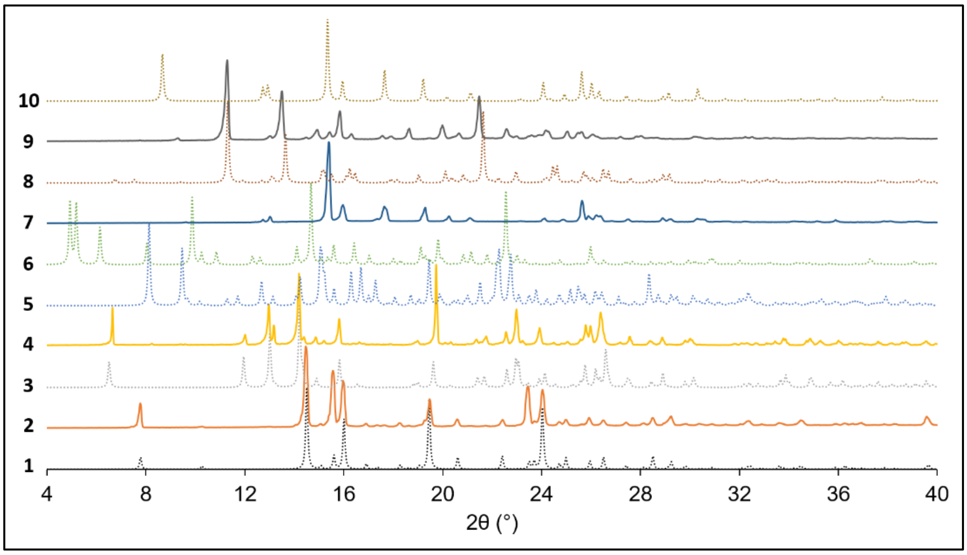


**Figure S33.** XRPD pattern of **1**: Nefiracetam FI (simulated), **2**: Nefiracetam after 7 days at 100%RH exposure, **3**: **NZC** (simulated), **4**: **NZC** (30 days at 100%RH), **5**: **NZCW** (simulated), **6**: **NCA** (simulated), **7**: **NCA** (30 days at 100%RH), **8**: **NOA** (simulated), **9**: **NOA** (30 days at 100%RH) and **10**: Nefiracetam monohydrate (simulated).

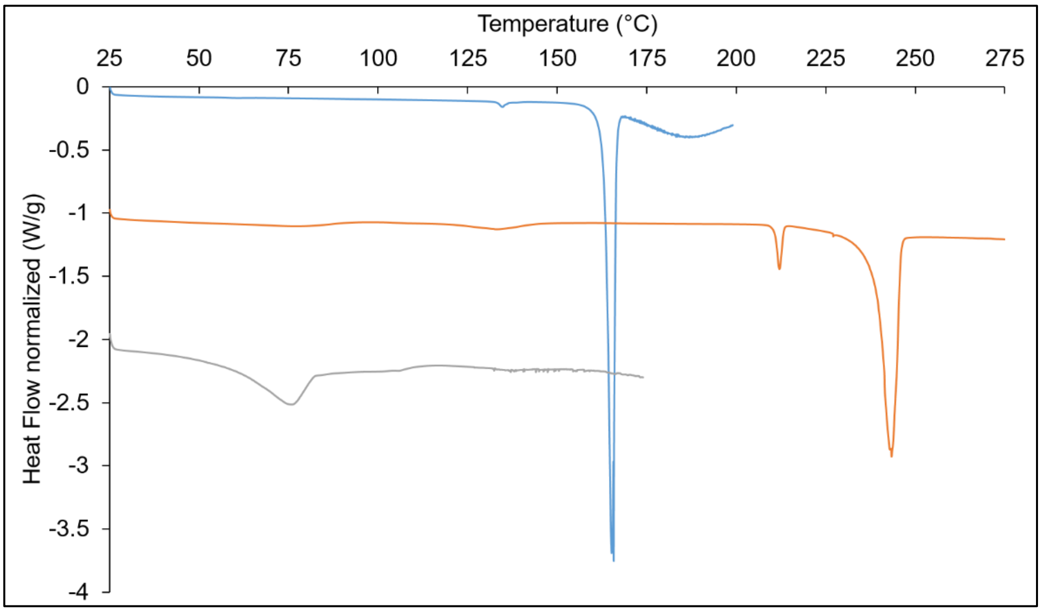


**Figure S34.** DSC curve of **NOA** (blue), **NZC** (orange) and **NCA** (grey) after 30 days under 100% RH exposure.

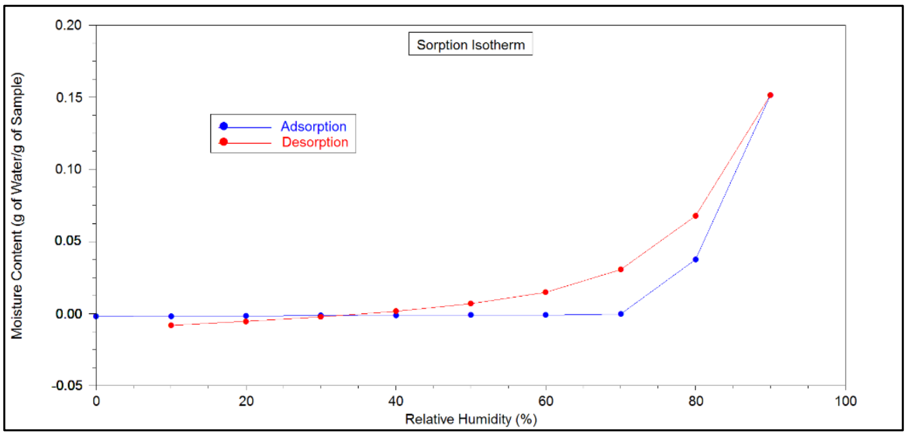


**Figure S35.** Sorption Isotherm (adsorption and desorption) of **NCA** at 25°C.

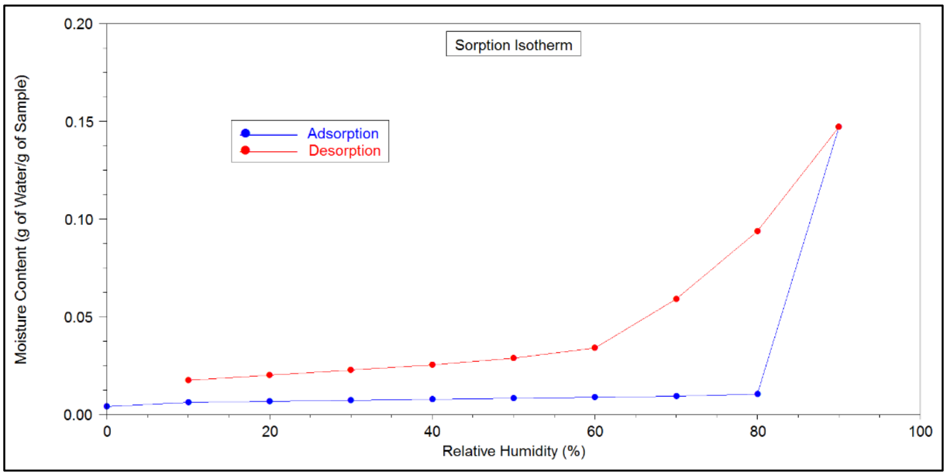


**Figure S36.** Sorption Isotherm (adsorption and desorption) of **NZC** at 25°C.

## Dissolution Experiments

*Calibration lines (HPLC and UVS)*

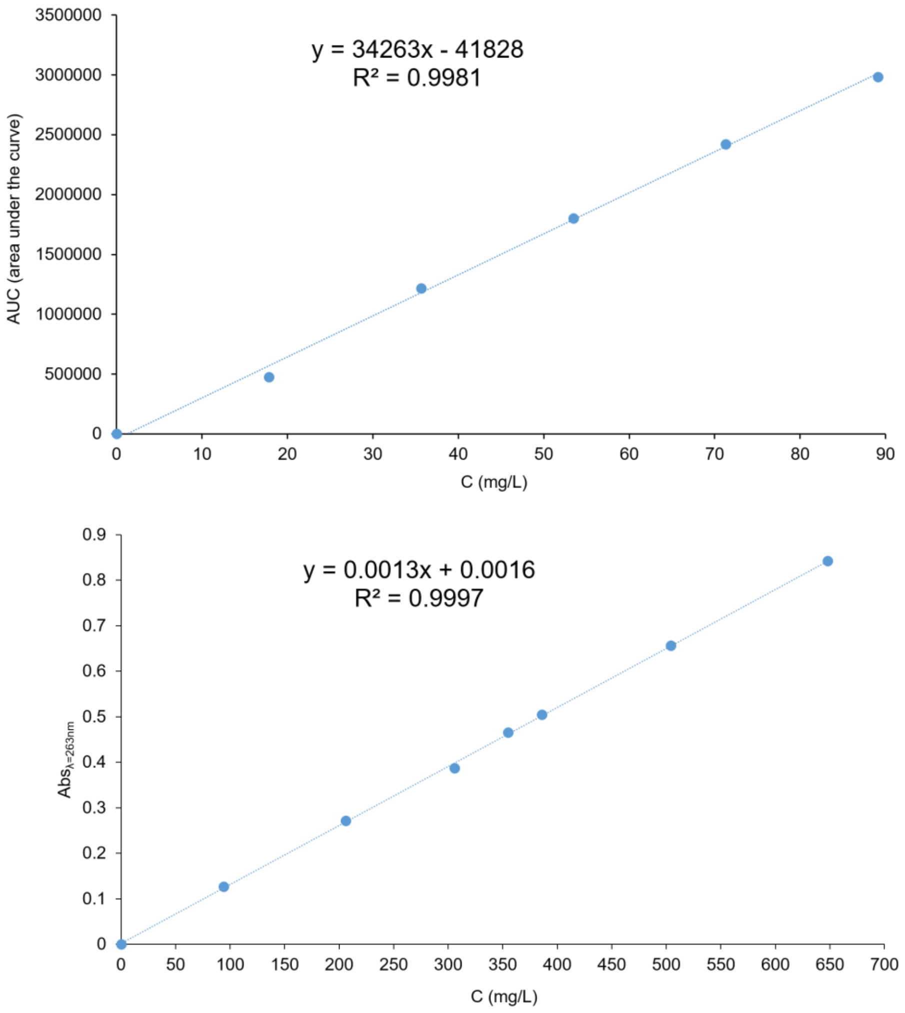


**Figure S37.** (top) Calibration line used to dose Nefiracetam in solution via HPLC during the dissolution experiments of **NCA** and **NOA** in EtOH and MeCN at 18°C. (below) Calibration line used to dose Nefiracetam in solution via UV spectroscopy during the dissolution experiments of **NZC** in EtOH and MeCN at 18°C.

*HPLC data*

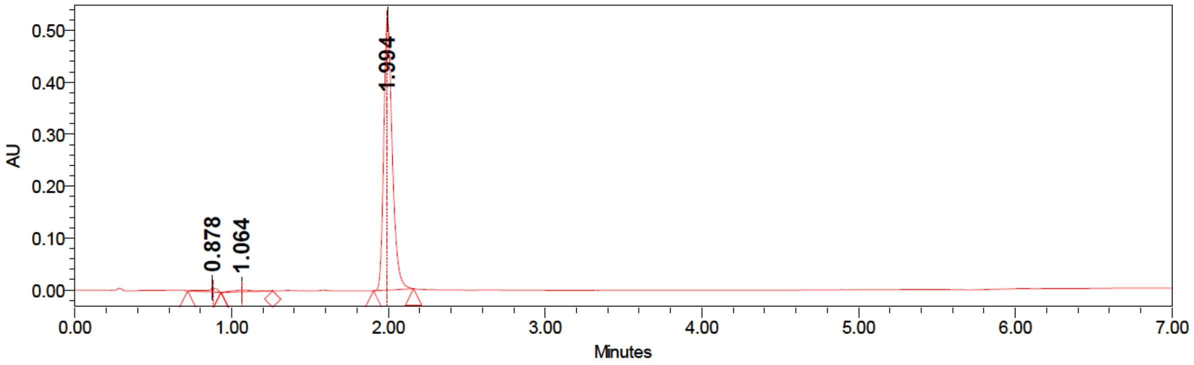


**Figure S38.** Typical HPLC chromatogram obtained during the quantitative analysis of Nefiracetam from the dissolution experiments.

**Table S6.** Summary of the data used to build the dissolution curves of **Nefiracetam** in EtOH and MeCN at 18°C under 100 rpm.

| **NEFIRACETAM** | **ACETONITRILE** | | | **ETHANOL** | | |
|---|---|---|---|---|---|---|
| Sampling time (min) | Dilution factor | Retention time (min) | AUC | Dilution factor | Retention time (min) | AUC |
| 0 | 0 | 0 | 0 | 0 | 0 | 0 |
| 0.08333333 | 2000 | 2.014 | 412097 | 2500 | 2.022 | 599373 |
| 0.16666667 | | | | | | |
| 0.25 | | | | | | |
| 0.33333333 | 2000 | 1.994 | 1123353 | 2500 | 2.003 | 891775 |
| 0.41666667 | | | | | | |
| 0.5 | 2000 | 1.994 | 1221140 | | | |
| 0.58333333 | | | | 2500 | 2.001 | 1430751 |
| 0.66666667 | 2000 | 1.993 | 1360187 | | | |
| 0.75 | | | | | | |
| 0.83333333 | | | | 2500 | 2.001 | 1660412 |
| 1 | 2000 | 1.998 | 1290697 | | 2.030 | 1803989 |
| 1.5 | 2000 | 1.999 | 1443300 | | | |
| 2 | 2000 | 1.998 | 1233092 | 2000 | 1.994 | 1903925 |
| 2.5 | 2000 | 1.998 | 1361664 | | | |
| 3 | 2000 | 2.000 | 1021522 | | | |
| 3.5 | | | | | | |
| 4 | 2000 | 1.998 | 1401631 | 2000 | 1.996 | 1810678 |
| 4.5 | | | | | | |
| 5 | 2000 | 1.998 | 1258620 | 2000 | 1.994 | 2058476 |
| 6 | | | | 2000 | 1.994 | 1975186 |
| 7 | | | | 2000 | 1.992 | 1797448 |
| 8 | | | | 2000 | 1.994 | 1975222 |
| 9 | | | | 2000 | 1.996 | 1688667 |
| 10 | 2000 | 1.996 | 1402725 | 2000 | 1.994 | 1994076 |
| 11 | | | | 2000 | 1.994 | 1781480 |
| 12 | | | | 2000 | 1.995 | 2119195 |
| 12.5 | | | | 2000 | 1.994 | 1980331 |
| 13 | | | | | | |
| 14 | | | | 2000 | 1.995 | 2200488 |
| 15 | 2000 | 1.998 | 1333962 | 2000 | 1.995 | 2191115 |
| 30 | 2000 | 1.997 | 1364834 | 2000 | 1.992 | 1746601 |
| 45 | | | | | | |
| 60 | 2000 | 2.001 | 1438496 | 2000 | 1.995 | 1775440 |
| 120 | | | | 2000 | 1.994 | 1653944 |
| 180 | 2000 | 2.000 | 1257201 | | | |
| 240 | | | | | | |
| 1440 | 2500 | 1.998 | 1141196 | 4000 | 2.039 | 1030530 |
| 1440 | 2500 | 2.027 | 1233540 | 4000 | 2.007 | 1052120 |
| 1440 | 2500 | 2.000 | 1181260 | 4000 | 2.007 | 1052120 |

**Table S7.** Summary of the data used to build the dissolution curves of **NCA** in EtOH and MeCN at 18°C under 100 rpm.

| **NCA** | **ACETONITRILE** | | | **ETHANOL** | | |
|---|---|---|---|---|---|---|
| Sampling time (min) | Dilution factor | Retention time (min) | AUC | Dilution factor | Retention time (min) | AUC |
| 0 | 0 | 0 | 0 | 0 | 0 | 0 |
| 0.08333333 | | | | | | |
| 0.16666667 | 2000 | 2.024 | 463252 | | | |
| 0.25 | | | | 4000 | 2.063 | 273133 |
| 0.33333333 | | | | | | |
| 0.41666667 | | | | | | |
| 0.5 | 2000 | 1.991 | 537961 | 4000 | 2.030 | 314596 |
| 0.58333333 | | | | | | |
| 0.66666667 | | | | | | |
| 0.75 | 2000 | 1.991 | 621607 | 4000 | 2.025 | 327294 |
| 0.83333333 | 2000 | 1.991 | 640564 | | | |
| 1 | | | | 4000 | 2.028 | 402443 |
| 1.5 | 2000 | 1.990 | 1016079 | 4000 | 2.030 | 767192 |
| 2 | 2000 | 1.990 | 1038334 | 4000 | 2.030 | 1303657 |
| 2.5 | 2000 | 1.990 | 864520 | 4000 | 2.029 | 1256000 |
| 3 | 2000 | 1.991 | 1046076 | 4000 | 2.031 | 1279776 |
| 3.5 | 2000 | 1.989 | 1059647 | 4000 | 2.029 | 1366063 |
| 4 | 2000 | 1.991 | 1038926 | 4000 | 2.040 | 1292413 |
| 4.5 | 2000 | 1.991 | 1109477 | 4000 | 2.030 | 1463559 |
| 5 | 2000 | 1.991 | 1171218 | 4000 | 2.031 | 1462498 |
| 6 | 2000 | 1.990 | 1083436 | | | |
| 7 | 2000 | 1.991 | 1170866 | 4000 | 2.031 | 1351450 |
| 8 | 2000 | 1.989 | 1166090 | 4000 | 2.031 | 1400703 |
| 9 | 2500 | 1.993 | 914513 | 4000 | 2.029 | 1330640 |
| 10 | 2500 | 1.992 | 873151 | 4000 | 2.033 | 1038031 |
| 11 | | | | | | |
| 12 | | | | | | |
| 12.5 | 2500 | 1.991 | 945260 | | | |
| 13 | | | | | | |
| 14 | | | | | | |
| 15 | 2500 | 1.994 | 886622 | 5000 | 2.032 | 1207073 |
| 30 | 2500 | 1.997 | | | 2.028 | 1134123 |
| 45 | | | | | | |
| 60 | 2500 | 1.996 | 1002506 | 5000 | 2.029 | 1198447 |
| 120 | 2500 | 1.998 | 1003643 | 5000 | 2.031 | 1293977 |
| 180 | | | | | | |
| 240 | | | | 5000 | 2.030 | 1247780 |
| 1440 | 2500 | 1.998 | 1063223 | 4000 | 2.040 | 1596102 |
| 1440 | 2500 | 1.997 | 1046565 | 4000 | 2.008 | 1558081 |
| 1440 | 2500 | 1.999 | 1034129 | 4000 | 2.008 | 1664379 |

**Table S8.** Summary of the data used to build the dissolution curves of **NOA** in EtOH and MeCN at 18°C under 100 rpm.

| NOA | ACETONITRILE | | | ETHANOL | | |
|---|---|---|---|---|---|---|
| **Sampling time (min)** | **Dilution factor** | **Retention time (min)** | **AUC** | Dilution factor | Retention time (min) | AUC |
| 0 | 0 | 0 | 0 | 0 | 0 | 0 |
| 0.08333333 | | | | | | |
| 0.16666667 | | | | 2500 | 2.035 | 695955 |
| 0.25 | 2000 | 2.147 | 479717 | | | |
| 0.33333333 | | | | | | |
| 0.41666667 | | | | 2500 | 2.003 | 577502 |
| 0.5 | 2000 | 2.111 | 603500 | | | |
| 0.58333333 | | | | | | |
| 0.66666667 | | | | | | |
| 0.75 | 2000 | 2.108 | 773451 | 2500 | 2.006 | 2140568 |
| 0.83333333 | | | | | | |
| 1 | 2000 | 2.106 | 785345 | 2500 | 2.003 | 596136 |
| 1.5 | 2000 | 2.107 | 692512 | 2500 | 2.004 | 826694 |
| 2 | 2000 | 2.105 | 822251 | 2500 | 2.005 | 905026 |
| 2.5 | 2000 | 2.104 | 872370 | 2500 | 2.004 | 2202520 |
| 3 | 2000 | 2.102 | 708751 | 2500 | 2.003 | 533911 |
| 3.5 | 2000 | 2.103 | 848988 | 2500 | 1.999 | 2307427 |
| 4 | 2000 | 2.099 | 796181 | | | |
| 4.5 | 2000 | 2.098 | 818237 | 2500 | 1.994 | 2394271 |
| 5 | 2000 | 2.096 | 861858 | 2500 | 2.000 | 2233971 |
| 6 | 2000 | 2.097 | 853860 | 2500 | 1.998 | 2359543 |
| 7 | 2000 | 2.095 | 851519 | 2500 | 1.998 | 2233371 |
| 8 | 2500 | 2.093 | 823877 | 2500 | 1.998 | 2211001 |
| 9 | 2500 | 2.094 | 661151 | 2500 | 1.997 | 2221166 |
| 10 | | | | | 1.998 | 2276022 |
| 11 | | | | | | |
| 12 | | | | | | |
| 12.5 | | | | 2500 | 1.998 | 2268118 |
| 13 | | | | | | |
| 14 | | | | | | |
| 15 | 2500 | 2.091 | 667857 | 2500 | 1.998 | 2290064 |
| 30 | 2500 | 2.092 | 651070 | 2500 | 1.994 | 2399600 |
| 45 | | | | | | |
| 60 | 2500 | 2.092 | 723930 | 2500 | 1.997 | 2360694 |
| 120 | 2500 | 2.090 | 652198 | 4000 | 1.997 | 2496967 |
| 180 | | | | | | |
| 240 | 2500 | 2.089 | 645758 | | | |
| 1440 | 2500 | 2.085 | 682654 | 4000 | 1.997 | 1534584 |
| 1440 | 2500 | 2.007 | 688118 | 4000 | 2.000 | 1500012 |
| 1440 | 2500 | 2.003 | 681328 | 4000 | 1.998 | 1508041 |

UVS data

**Table S9.** Summary of the data used to build the dissolution curves of **NZC** in EtOH and MeCN at 18°C under 100 rpm.

| NZC | ACETONITRILE | | | ETHANOL | | |
|---|---|---|---|---|---|---|
| **Sampling time (min)** | **Dilution factor** | **Retention time (min)** | **Abs263** | Dilution factor | Retention time (min) | Abs263 |
| 0 | | - | | | - | |
| 0.08333333 | | - | | | - | |
| 0.16666667 | 250 | - | 0.0278 | 250 | - | 0.1135 |
| 0.25 | | - | | | - | |
| 0.33333333 | | - | | | - | |
| 0.41666667 | 250 | - | 0.0278 | 250 | - | 0.1350 |
| 0.5 | | - | | | - | |
| 0.58333333 | | - | | | - | |
| 0.66666667 | | - | | 250 | - | 0.1608 |
| 0.75 | | - | | | - | |
| 0.83333333 | 250 | - | 0.0314 | | - | |
| 1 | | - | | 250 | - | 0.1359 |
| 1.5 | 250 | - | 0.033 | 250 | - | 0.1544 |
| 2 | 250 | - | 0.0331 | 250 | - | 0.1036 |
| 2.5 | 250 | - | 0.0337 | 250 | - | 0.1237 |
| 3 | | - | | 250 | - | 0.1036 |
| 3.5 | 250 | - | 0.0341 | 250 | - | 0.1310 |
| 4 | 250 | - | 0.0376 | 250 | - | 0.1310 |
| 4.5 | 250 | - | 0.0374 | 250 | - | 0.1207 |
| 5 | 250 | - | 0.0342 | 250 | - | 0.1859 |
| 6 | 250 | - | 0.0328 | | - | |
| 7 | 250 | - | 0.0331 | 250 | - | 0.1517 |
| 8 | 250 | - | 0.0333 | 250 | - | 0.1494 |
| 9 | 250 | - | 0.0326 | 250 | - | 0.1652 |
| 10 | 250 | - | 0.0337 | 250 | - | 0.1669 |
| 11 | | - | | | - | |
| 12 | | - | | | - | |
| 12.5 | 250 | - | 0.0337 | | - | |
| 13 | | - | | | - | |
| 14 | | - | | | - | |
| 15 | 250 | - | 0.0337 | 250 | - | 0.1451 |
| 30 | | - | | | - | |
| 45 | | - | | | - | |
| 60 | 250 | - | 0.0333 | | - | |
| 120 | 250 | - | 0.0333 | | - | |
| 180 | | - | | | - | |
| 240 | | - | | | - | |
| 1440 | 250 | - | 0.0342 | 250 | - | 0.2590 |
| 1440 | 250 | - | 0.0334 | 250 | - | 0.2573 |
| 1440 | 250 | - | 0.035 | 250 | - | 0.2549 |
